# Short-Term Effects of Targeted Movement Training on Gait Kinematics in Children with Juvenile Idiopathic Arthritis: A Motion Analysis Study

**DOI:** 10.3390/jcm15103650

**Published:** 2026-05-09

**Authors:** Sibel Özbal, Asya Albayrak, Asena Yekdaneh, İrem Dönmez, Nuray Aktay Ayaz, Nilay Arman, Hande Argunsah

**Affiliations:** 1 Department of Biomedical Engineering, Graduate School of Natural and Applied Sciences, Acibadem Mehmet Ali Aydinlar University, Istanbul 34752, Turkey; sibel.ozbal@live.acibadem.edu.tr; 2 Department of Physiotherapy and Rehabilitation, Faculty of Health Sciences, Istanbul Kent University, Istanbul 34406, Turkey; asiaalbayrak@gmail.com; 3 Program of Physiotherapy, Vocational School of Health Services, Fenerbahçe University, Istanbul 34758, Turkey; asenayekdaneh@gmail.com; 4 Physiotherapy and Rehabilitation Master of Science Program, Institute of Graduate Studies, Istanbul University-Cerrahpasa, Istanbul 34320, Türkiye; iremdnmzz78@gmail.com; 5 Division of Pediatric Rheumatology, Department of Pediatrics, Istanbul Faculty of Medicine, Istanbul University, Istanbul 34093, Türkiye; 6 Department of Physiotherapy and Rehabilitation, Faculty of Health Sciences, Istanbul University-Cerrahpasa, Istanbul 34320, Turkey; nilayarman@iuc.edu.tr; 7 Department of Biomedical Engineering, Faculty of Engineering and Natural Sciences, Acibadem Mehmet Ali Aydinlar University, Istanbul 34752, Turkey

**Keywords:** juvenile idiopathic arthritis, wearable motion capture, movement training, pediatric rehabilitation, trunk stability, motor control

## Abstract

**Background**: Children with juvenile idiopathic arthritis (JIA) exhibit gait abnormalities, postural instability, and compensatory movement strategies due to joint pain, inflammation, and reduced neuromuscular control. These alterations negatively affect functional mobility and movement efficiency. Although gait retraining is commonly recommended in rehabilitation, objective evidence on its short-term biomechanical effects remains limited. This study aimed to evaluate the immediate impact of a single-session standardized movement training intervention on gait biomechanics in children with JIA. **Methods**: Seventeen children with JIA underwent pre–post gait assessments using the Xsens MVN Awinda wearable motion capture system. The intervention focused on step symmetry, stride length, heel–toe progression, and upright trunk posture, delivered by an experienced physiotherapist following a standardized protocol. Scalar kinematic outcomes were analyzed using paired statistical tests, and time-normalized kinematic waveforms were compared with healthy reference data from 25 age-matched participants derived from the COMPWALK-ACL dataset. **Results**: Significant improvements were observed in multiple gait parameters following the intervention. Trunk lateral lean decreased significantly (*p* = 0.0002; d = −1.35), indicating enhanced postural stability. Significant changes were also found in ankle dorsiflexion–plantarflexion (*p* = 0.0081; d = 0.83) and knee flexion–extension (*p* = 0.0252; d = 0.68). Waveform analyses showed increased similarity to healthy patterns, particularly in trunk and knee kinematics. Spatiotemporal parameters reflected a slower, more controlled gait pattern, with increased stride time and stance duration. **Conclusions**: A single session of standardized movement training can produce immediate improvements in gait biomechanics in children with JIA, especially in trunk control and lower-limb kinematics. Wearable motion analysis provides a sensitive tool for detecting these short-term adaptations and supports the inclusion of structured movement training in pediatric JIA rehabilitation.

## 1. Introduction

Children with juvenile idiopathic arthritis (JIA) frequently exhibit altered biomechanics and impairments in physical function due to joint pain, stiffness, and muscle weakness [1,2,3]. These impairments result in decreased participation in physical activity, delayed motor development, and diminished quality of life [4,5]. Gait abnormalities, balance deficits, and functional limitations such as difficulty in sit-to-stand transitions or single-leg stance are commonly reported [6,7,8].

This condition often results in persistent joint inflammation, muscle weakness, and pain, which can impair postural control and gait function. These children frequently present with asymmetrical movement patterns, disturbed balance, altered trunk orientation, and asymmetries in upper body alignment. Such biomechanical deviations may cause joint stress, increase fatigue, and impair participation in daily activities. While exercise interventions targeting balance and functional mobility are recommended, there is limited quantitative evidence describing their immediate biomechanical effects in pediatric rheumatology populations. Despite pharmacologic advances, there remains a need for non-pharmacological interventions that target motor function directly.

Movement training programs focusing on motor learning principles and task-specific training have been shown to improve functional mobility and sensorimotor control in various pediatric populations [9,10,11]. Such programs typically include structured verbal instruction, therapist demonstration, and guided practice targeting key spatiotemporal and kinematic components of gait. These components often involve improving step symmetry and stride length to enhance temporal coordination and movement efficiency, promoting heel–toe progression to support appropriate foot loading and push-off mechanics, and encouraging upright trunk posture to optimize postural control and balance during walking. By addressing these elements, movement training aims to modify both the timing and coordination of gait as well as segmental alignment and movement quality. A recent systematic review emphasized the lack of motion analysis-based trials assessing the effectiveness of physiotherapy interventions in children with rheumatic conditions [12]. In this context, gait analysis typically focuses on key biomechanical parameters such as trunk orientation, which reflects postural control; center of mass (CoM) displacement patterns, which indicate dynamic stability and balance strategies; lower extremity joint range of motion (ROM), which represents joint-level movement capacity and coordination; and shoulder orientation, which provides insight into upper-body movement and arm swing coordination during walking. These parameters collectively enable a comprehensive evaluation of gait adaptations at both segmental and whole-body levels. The integration of wearable motion capture technologies enables detailed quantification of joint kinematics and postural control in naturalistic settings, offering new opportunities to assess training-related changes [13,14,15]. In addition to their role in objective measurement, wearable motion analysis systems are highly sensitive to detecting subtle and immediate changes in movement patterns. This capability is especially relevant in pediatric and clinical populations, where early motor adaptations may not be captured through conventional observational or clinical assessments. Therefore, the use of wearable motion analysis in the present study allows for a more precise evaluation of immediate biomechanical responses to targeted movement training.

The aim of this study was to evaluate the immediate effects of a single-session movement training intervention (approximately 30–40 min) on gait biomechanics, with outcomes assessed immediately post-intervention in children with JIA using wearable motion analysis technology. By quantifying biomechanical improvements following a standardized intervention, this study aimed to provide objective evidence. This may support the inclusion of structured motor training in pediatric rheumatology rehabilitation protocols.

## 2. Materials and Methods

### 2.1. Participants

This prospective single-session pre–post intervention study was conducted in a pediatric rheumatology outpatient setting. A total of 17 pediatric patients diagnosed with JIA (7 females, 10 males; mean age: 13.6 ± 2.8 years). JIA patients were included based on functional mobility criteria rather than JIA subtype classification. All included patients were able to walk independently, suggesting functional involvement of the lower extremities sufficient to influence gait performance. Detailed information regarding medication use was not systematically recorded. All participants were clinically stable and not experiencing an active disease flare at the time of assessment.

A total of 25 age-matched healthy controls (10 females, 15 males; mean age: 15.7 ± 0.6 years) were included in the study (Table 1). A healthy pediatric control group was derived from the publicly available COMPWALK-ACL dataset [16] (Table 1). This dataset includes biomechanical gait data collected from typically developing children with no reported history of neurological, musculoskeletal, or orthopedic disorders affecting gait. Only participants meeting the specified age range were included in the control group analysis. The COMPWALK-ACL dataset was used exclusively for comparative purposes to provide normative reference values for gait-related outcome measures. All data were fully anonymized and publicly accessible. A priori power analysis was conducted using G*Power (version 3.2.1, Universität Düsseldorf, Germany) to determine the minimum sample size required for a two-tailed paired *t*-test (within-subjects design), assuming α = 0.05, power (1 − β) = 0.95, and a moderate-to-large effect size of d = 0.75.

Between-group comparisons demonstrated that the control group was significantly older (*p* = 0.010) and taller (*p* < 0.001) than the JIA group. No statistically significant differences were observed between groups in body weight (*p* = 0.132), body mass index (*p* = 0.627), or sex distribution (*p* = 1.000).

Participants were eligible for inclusion if they had a clinical diagnosis of JIA for at least six months, were able to stand and walk independently without assistive devices, had no cognitive or sensory impairments, and had not undergone any surgical interventions or intra-articular injections within the previous three months. Children were excluded if they had severe lower limb joint deformities or fixed contractures, were experiencing an active systemic disease flare during the assessment period or used orthotic devices that limited full range of motion.

Informed consent was obtained from the legal guardians of all participants, and child assent was obtained in accordance with the Declaration of Helsinki. Data collection was conducted between 8 February 2026 and 1 March 2026. The study was approved by the Clinical Research Ethics Committee (protocol code: 02.2026FBU; approval date: 20 January 2026).

### 2.2. Study Protocol

A pre–post experimental design was employed for the JIA group. At baseline, participants performed the walking task without any prior instruction (pre-training condition). Following completion of the movement training intervention, participants were re-assessed under the post-training condition.

Each participant underwent a single-session intervention, lasting approximately 30–40 min, followed by immediate post-intervention assessment. Training intervention delivered using a fully standardized protocol designed to optimize gait mechanics. The intervention was administered individually by the same experienced physiotherapist in a controlled clinical environment to eliminate inter-therapist variability.

The training targeted four predefined spatiotemporal and kinematic components of gait: step symmetry, stride length, heel–toe progression, and upright trunk posture. Instruction followed a structured motor-learning framework consisting of standardized verbal explanations, therapist demonstration, and guided practice. The content, sequence, task order, and duration of the intervention (approximately 30–40 min) were identical for all participants, and uniform criteria were used to define correct performance.

Although real-time verbal feedback was provided during practice, it was strictly limited to reinforcing the predefined target gait pattern and did not modify the intervention content, progression, or duration. This approach ensured a standardized and reproducible training protocol across participants while allowing sufficient practice for all participants to achieve the predefined movement pattern and enabling objective evaluation of pre- to post-training changes in gait mechanics.

Healthy control data were obtained from the publicly available COMPWALK-ACL dataset, which comprises biomechanical gait recordings during normal walking. Control participants performed walking trials in a single session without receiving any training or feedback, serving as a normative reference for natural gait patterns.

Kinematic data for both groups were recorded using the Xsens Technologies B.V. MVN Awinda motion capture system (Enschede, The Netherlands), which consists of 17 inertial measurement units (IMUs) positioned on standardized anatomical landmarks. Participants performed a 5 m walking task at a self-selected comfortable pace in a controlled laboratory environment. A 5 m walking task was selected to ensure feasibility in a pediatric rheumatology outpatient setting and to minimize fatigue or discomfort during repeated pre–post assessments. Extracted gait parameters included trunk orientation, center of mass (CoM) displacement patterns, lower extremity joint range of motion (ROM), and shoulder orientation during walking.

**Figure 1:**
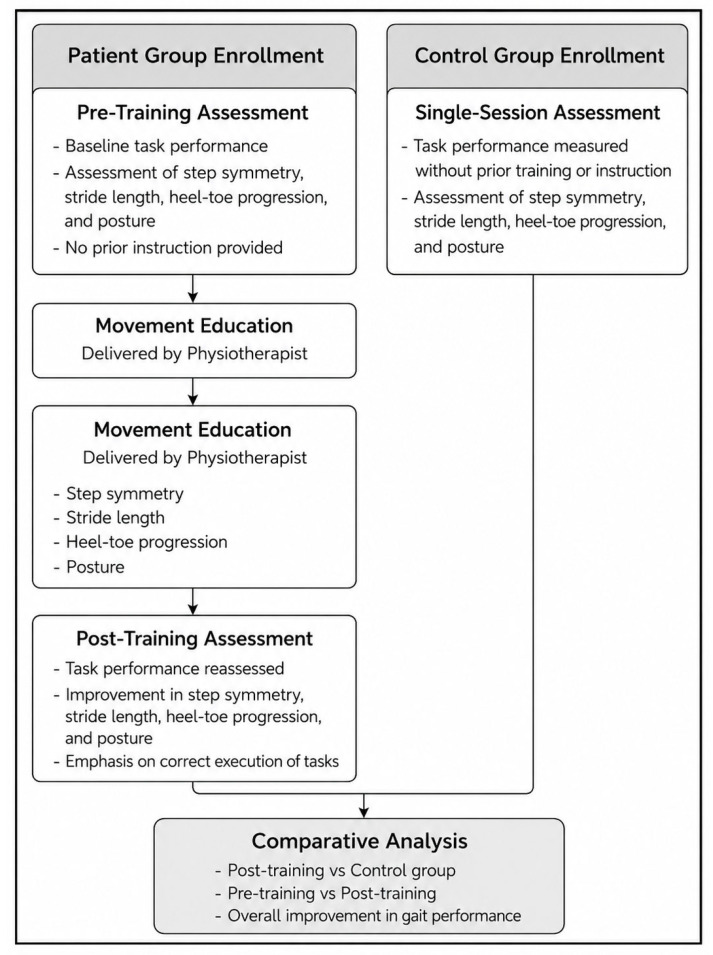
Flowchart illustrating the study design and experimental protocol for the patient and control groups, including pre-training assessment, movement education-based gait training, post-training assessment, and comparative analysis.

For the JIA group, all parameters were obtained both before and immediately after the intervention to enable within-subject pre–post comparisons. Control group data were used exclusively for between-group comparisons to contextualize patient gait characteristics relative to normative pediatric walking patterns (Figure 1).

### 2.3. Statistical Analysis

All analyses were performed using IBM SPSS Statistics (version 29; IBM Corp., Armonk, NY, USA). Spatiotemporal and kinematic parameters are reported as mean ± standard deviation.

For the JIA group, a within-subject pre–post experimental design was employed to examine immediate adaptations following a single session of standardized movement training. Spatiotemporal and CoM parameters were analyzed descriptively, with emphasis on the magnitude and direction of pre- to post-training changes and associated changes in variability.

Time-normalized kinematic waveforms were analyzed across the gait cycle using waveform similarity metrics, including root mean square error (RMSE) and mean absolute difference (MAD), to quantify both the magnitude of pre–post waveform adaptations and the distance of patient trajectories from healthy control patterns. Changes in waveform similarity were expressed as percentage differences, indicating whether post-training kinematics shifted toward or away from normative gait profiles. Kinematic data from the healthy control group were used exclusively as a normative reference to contextualize patient gait characteristics.

For subject-level scalar kinematic outcomes, the normality of pre–post difference scores was assessed using the Shapiro–Wilk test. Parameters meeting normality assumptions were compared using paired-samples *t*-tests, whereas Wilcoxon signed-rank tests were applied when normality was violated. Effect sizes were quantified using paired Cohen’s d with 95% confidence intervals, and statistical significance was set at *p* < 0.05. Control group data were not included in inferential statistical testing.

## 3. Results

The results characterize immediate gait adaptations following a single session of standardized movement training in the JIA group. Within-subject comparisons focus on pre- to post-training changes in spatiotemporal parameters and kinematic gait profiles to quantify training-related effects. Kinematic waveforms of the lower limbs, pelvis, trunk, and upper extremities were examined across the normalized gait cycle to identify phase-specific adaptations and changes in movement consistency. Data from age-matched healthy controls were included solely as a normative reference to contextualize patient gait characteristics relative to typical pediatric walking patterns.

**Figure 2:**
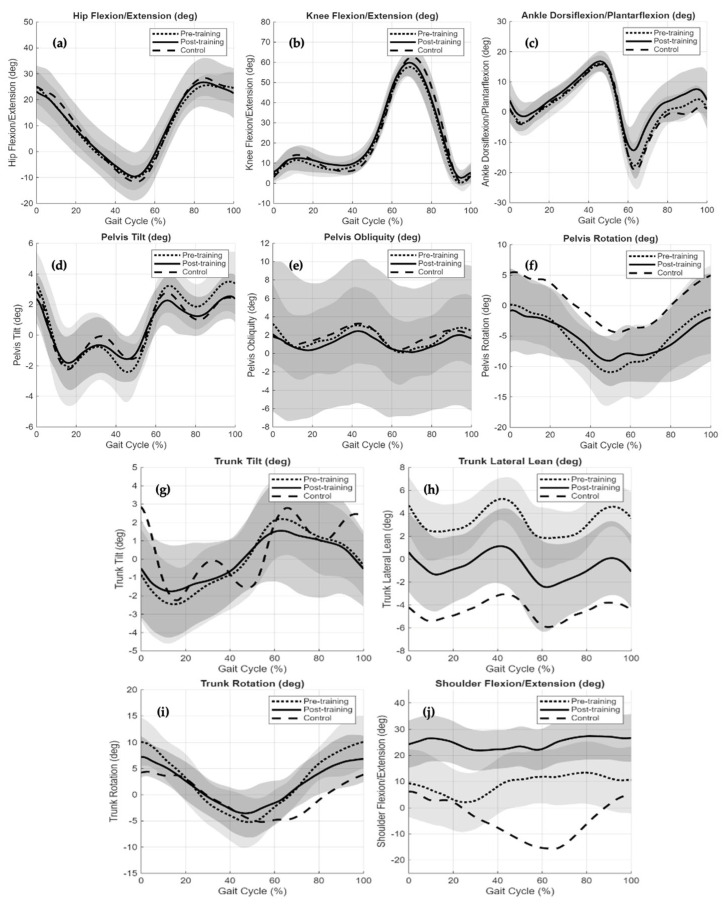
Averaged sagittal and frontal plane kinematics across the normalized gait cycle (0–100%) for the JIA group before training (pre-training), after training (post-training), and healthy controls. Solid and dashed lines represent group mean trajectories, and shaded bands indicate ±1 standard deviation. (**a**) Hip Flexion/Extension (deg), (**b**) Knee Flexion/Extension (deg), (**c**) Ankle Dorsiflexion/Plantarflexion (deg), (**d**) Pelvis Tilt (deg), (**e**) Pelvis Obliquity (deg), (**f**) Pelvis Rotation (deg), (**g**) Trunk Tilt (deg), (**h**) Trunk Lateral Lean (deg), (**i**) Trunk Rotation (deg), (**j**) Shoulder Flexion/Extension (deg).

Figure 2 illustrates phase-dependent changes in lower-limb, pelvic, trunk, and upper-limb kinematics across the gait cycle following the standardized movement training intervention. Overall, post-training trajectories demonstrated reduced waveform variability and altered segmental coordination, particularly at the pelvis and trunk, indicating short-term improvements in movement consistency and postural control.

At the lower-extremity level (Figure 2a–c), hip, knee, and ankle flexion–extension profiles preserved their characteristic gait-cycle morphology after training. Post-training curves largely overlapped pre-training trajectories, maintaining similar peak timing and overall excursion patterns. However, reduced variability was observed, especially during stance and push-off phases. These findings suggest that joint-level movement patterns were preserved while consistency of execution improved.

Pelvic kinematics (Figure 2d–f) exhibited clearer post-training adaptations. Pelvic tilt and rotation demonstrated reduced excursion amplitudes and smoother transitions between gait phases. Post-training profiles appeared more aligned with control trajectories, particularly during mid-stance and terminal stance. Changes in pelvic obliquity were less pronounced but showed a trend toward reduced oscillatory behaviour across the gait cycle.

The most prominent modifications were observed in trunk kinematics (Figure 2g–i). Trunk tilt and lateral lean displayed visibly reduced amplitudes and attenuated oscillations following training. These reductions were especially evident during single-limb support, where trunk motion appeared more regulated. Trunk rotation also demonstrated decreased excursion and reduced variability, indicating improved coordination between trunk and pelvic segments during walking.

Upper-limb kinematics (Figure 2j) further reflected post-training adaptations. Shoulder flexion–extension trajectories showed reduced arm swing amplitude compared with pre-training, with post-training patterns approaching the magnitude and temporal organization observed in healthy controls. Although post-training waveforms did not fully converge with control patterns across all variables, the most consistent adaptations were observed in proximal and trunk-related segments. Collectively, these findings demonstrate immediate modifications in segmental coordination and movement consistency across the gait cycle following the standardized movement training intervention.

Table 2 presents waveform similarity metrics comparing gait kinematics of children with JIA before and after the movement training intervention relative to healthy controls. Similarity was quantified using RMSE and MAD between patient and control waveforms, where lower values indicate greater similarity to the healthy reference pattern.

The intervention produced notable improvements in several trunk-related kinematic parameters. The largest improvements were observed for trunk lateral lean, trunk rotation, and trunk tilt, corresponding to approximately 43% improvement relative to the control waveform. These findings suggest that the movement training program was particularly effective in improving upper-body postural control and trunk stabilization during gait.

A moderate improvement was observed in knee flexion–extension kinematics (12.26% improvement), while MAD showed a small reduction (2.36%). This indicates that the training may have modestly improved sagittal-plane knee mechanics during gait, although the magnitude of change was smaller compared with trunk parameters.

In contrast, several parameters showed minimal change or slight deterioration relative to the control waveform, including pelvis tilt, pelvis rotation, pelvis obliquity, and hip flexion–extension. These variables demonstrated small increases in RMSE and MAD after training, suggesting that the short-term intervention had limited influence on pelvic alignment and hip kinematics.

More pronounced divergence from control patterns was observed for shoulder flexion–extension and ankle dorsiflexion–plantarflexion. Shoulder kinematics showed a substantial increase in RMSE and MAD, indicating greater deviation from the control waveform following training. Similarly, ankle kinematics demonstrated a marked increase in RMSE and MAD. These findings may reflect compensatory strategies adopted by participants as they attempted to modify their gait pattern during the brief training session.

Table 3 summarizes the subject-level changes in gait kinematic parameters following the targeted movement training session. Paired statistical comparisons revealed that several parameters demonstrated significant or meaningful changes after the intervention.

The most pronounced change was observed in trunk lateral lean, which showed a highly significant reduction following training with a large effect size. This finding indicates a decrease in lateral trunk displacement during gait and suggests improved frontal-plane trunk control and postural stability.

Significant changes were also observed in ankle dorsiflexion–plantarflexion and knee flexion–extension. These results indicate moderate-to-large improvements in sagittal-plane lower-limb mechanics following training. The improvement in ankle motion may reflect enhanced push-off control and improved foot progression during walking, while the change in knee flexion–extension suggests more effective shock absorption and propulsion mechanics.

In contrast, several other parameters did not demonstrate statistically significant changes. Variables related to pelvic motion (pelvis tilt, rotation, and obliquity) remained relatively stable following the intervention, with small effect sizes and confidence intervals that crossed zero. Similarly, CoM anterior–posterior excursion, mediolateral sway, and vertical oscillation did not show significant alterations, indicating that the intervention primarily influenced specific components of postural and joint control rather than global gait mechanics.

Changes in shoulder flexion–extension also did not reach statistical significance despite a moderate effect size. The wide confidence interval indicates substantial inter-individual variability in arm swing responses following training, suggesting that upper-limb coordination may be more variable and less directly influenced by the brief intervention.

Table 4 summarizes the spatiotemporal gait parameters and CoM measures before and after the standardized movement training intervention, with healthy control values provided as a normative reference. Following training, stride time increased, whereas cadence and walking speed decreased, indicating a shift toward a slower and more controlled gait pattern. Stride length showed a slight reduction, while step width demonstrated a modest increase. The temporal distribution of the gait cycle changed, with an increased proportion of stance phase and a corresponding reduction in swing phase. Double support duration also increased post-training. Upper-body kinematics exhibited marked changes, with a reduction in arm swing range of motion and decreased trunk lean amplitude, indicating improved control of frontal-plane trunk movement during gait. With respect to CoM dynamics, vertical oscillation remained stable across conditions. Mediolateral CoM displacement increased following training, whereas anterior–posterior excursion remained similar in magnitude, accompanied by reduced variability. Together, these findings demonstrate measurable modifications in spatiotemporal organization, upper-body kinematics, and CoM behaviour immediately following the standardized movement training intervention.

## 4. Discussion

This study investigated the immediate biomechanical effects of a single-session, standardized movement training intervention on gait performance in children with JIA using wearable motion analysis. The principal finding was a selective and statistically significant reduction in trunk lateral lean, accompanied by a moderate-to-large effect size, indicating improved frontal-plane trunk control during walking. Similar proximal adaptations following brief motor interventions have been reported in pediatric musculoskeletal and neurological populations, where postural control emerges as an early and sensitive marker of change [17,18,19,20]. This adaptation was further supported by waveform-level analyses demonstrating reduced variability and convergence toward normative patterns in trunk and proximal kinematics, consistent with prior work highlighting the value of time-series-based metrics for detecting subtle motor improvements. These findings suggest that even a brief, well-structured movement training session can elicit meaningful short-term adaptations in postural regulation in this population.

Among all subject-level scalar outcomes, trunk lateral lean emerged as the only parameter exhibiting a statistically significant pre–post change. This result is clinically and biomechanically relevant, as excessive frontal-plane trunk motion is commonly observed in children with JIA and is often interpreted as a compensatory strategy related to pain, joint instability, or reduced proximal control [21,22]. The observed reduction in trunk lateral lean, supported by both inferential statistics and waveform similarity metrics, indicates improved axial stability and more efficient load transfer during single-limb support, in line with previous gait analyses in pediatric rheumatology cohorts [23]. In the waveform similarity analysis, trunk lateral lean demonstrated a substantial reduction in distance to the healthy control waveform (RMSE reduction ≈ 43% and MAD reduction ≈ 43%), confirming that the post-training pattern more closely resembled normative gait trajectories. This finding aligns with the intervention’s emphasis on upright posture and controlled gait execution, suggesting that proximal postural mechanisms may be particularly responsive to short-term motor learning [24].

Waveform similarity analyses reinforced this interpretation, revealing substantial reductions in distance to healthy control patterns for trunk lateral lean, trunk rotation, and trunk tilt. These trunk-related parameters showed the largest improvements relative to the control waveform, indicating that the intervention primarily influenced proximal postural control mechanisms. Importantly, these improvements were characterized not only by changes in mean trajectory but also by reduced variability across the gait cycle, indicating enhanced movement consistency. Such consistency is increasingly recognized as a key marker of improved motor control and neuromuscular organization, particularly in pediatric populations with chronic musculoskeletal conditions [25,26]. Collectively, these findings suggest that the short-term movement training program effectively enhanced trunk stabilization and axial coordination during gait, likely improving the control of the body’s center of mass during single-limb support.

In contrast to trunk-dominant changes, lower-limb joint kinematics demonstrated more modest and variable responses. While knee flexion–extension exhibited moderate convergence toward the control waveform, reflected by reductions in RMSE and MAD, these changes did not reach statistical significance at the scalar level. Hip kinematics remained largely unchanged relative to the control pattern, and pelvic parameters such as pelvic tilt, rotation, and obliquity showed only minor deviations following the intervention. At the distal level, ankle dorsiflexion–plantarflexion waveforms diverged from normative trajectories after training. This dissociation suggests that short-term movement training preferentially influences proximal and postural control, while distal joint mechanics may require longer exposure, repeated practice, or task-specific strengthening to demonstrate normalization [27,28].

The divergence observed in ankle kinematics and shoulder motion should not be interpreted as deterioration. Rather, these changes may reflect a temporary reorganization of movement strategies, where reductions in trunk and arm compensations alter distal joint demands. Similar transient effects have been reported in early phases of motor retraining, where the central nervous system prioritizes stability and control over efficiency or symmetry [25,29]. In the present dataset, shoulder flexion–extension waveforms displayed greater deviation from control patterns following training, which may reflect altered arm swing coordination as participants attempted to maintain improved trunk alignment. Descriptive analysis of spatiotemporal and CoM parameters further indicated a consistent shift toward a slower and more controlled gait pattern following training, characterized by increased stride time, reduced cadence and speed, and modest increases in stance and double support phases. Such changes are consistent with a motor learning process in which participants prioritize accuracy and stability over velocity, particularly immediately after instruction [30].

From a clinical perspective, these findings support the inclusion of structured movement training as a feasible and effective component of pediatric rheumatology rehabilitation, even when delivered in a single session. The results suggest that brief targeted movement training may rapidly improve trunk stability during gait, which is a critical component of efficient locomotion in children with rheumatic conditions. However, the persistence of variability in ankle mechanics, pelvic motion, and upper-limb coordination indicates that additional training sessions or more targeted exercises may be required to achieve broader normalization of gait patterns relative to healthy children. Notably, while scalar outcomes identified statistically significant changes in trunk lateral lean, waveform analyses provided complementary insight into phase-dependent adaptations and system-level coordination, consistent with recent recommendations for multi-level biomechanical assessment in pediatric populations.

Several limitations should be acknowledged. First, the study evaluated only immediate post-training effects; therefore, the durability and retention of observed adaptations remain unknown. This design was intentionally chosen to isolate short-term motor learning responses to a single-session intervention and to minimize the influence of external or disease-related variability. However, the absence of follow-up assessments limits conclusions regarding long-term clinical impact. Future studies should include multi-session interventions and longitudinal follow-up to evaluate the persistence of these effects. The lack of detailed information on medication use represents an additional limitation, as pharmacological treatment may influence motor performance and gait characteristics. The heterogeneity of JIA subtypes and the lack of detailed classification of joint involvement represent additional limitations. Although all participants demonstrated sufficient lower extremity involvement to affect gait function, the absence of subtype-specific analysis limits the ability to generalize findings across different JIA phenotypes. In addition, the relatively short 5 m walking distance may restrict the generalizability of the findings to sustained walking performance. Although the same standardized distance was used for all pre- and post-training assessments, future studies should incorporate longer walking distances and repeated trials to improve the reliability of gait estimates. Importantly, the observed improvements should not be interpreted as evidence that a single-session intervention is sufficient to produce sustained or clinically meaningful long-term changes in gait. Rather, these findings reflect immediate motor adaptations following targeted instruction and should be considered as an initial step that may be reinforced through repeated and longer-term rehabilitation interventions.

## 5. Conclusions

A single session of standardized movement training induced immediate, targeted changes in gait biomechanics in children with JIA, particularly in trunk control. However, these changes reflect short-term adaptations, and their persistence over time remains unknown. Structured and repeated rehabilitation interventions are likely required to achieve sustained functional improvements. Wearable motion analysis provides an objective and sensitive approach to detect such immediate biomechanical adaptations, supporting more precise evaluation of intervention effects in clinical settings. Structured movement training represents a promising, low-burden strategy to support functional mobility in pediatric rheumatology care.

## Figures and Tables

**Table 1:** Demographic and clinical characteristics of patient and control groups (*p* < 0.05).

Parameter	JIA Group	Control Group	*p*-Value
Sample size (n)	17	25	–
Age (years)	13.7 ± 2.8	15.7 ± 0.7	0.010 †
Gender (M/F)	10/7	15/10	1.000 ‡
Height (cm)	156.9 ± 12.1	168.9 ± 7.5	<0.001 †
Weight (kg)	54.3 ± 15.6	60.7 ± 7.9	0.132 †
Body mass index (kg/m^2^)	21.7 ± 4.5	21.1 ± 2.3	0.627 †
Dominant side (R/L)	17/0	25/0	–
Diagnosis	JIA: 17	Healthy: 25	–

† Independent-samples *t*-test (Welch correction). ‡ Chi-square test. JIA: Juvenile Idiopathic Arthritis M: Male, F: Female; R: Right, L: left.

**Table 2:** Waveform Similarity Metrics for Pre- to Post-Training Gait Kinematics Relative to Healthy Controls.

Parameter (Deg)	RMSE to Control (Pre)	RMSE to Control (Post)	RMSE Change (Pre–Post)	MAD to Control (Pre)	MAD to Control (Post)	MAD Change (Pre–Post)	RMSE %	MAD %
Trunk Lateral Lean	3.507	2.001	−1.506	3.368	1.907	−1.461	42.93	43.38
Knee Flexion Extension	3.519	3.087	−0.432	2.732	2.667	−0.065	12.26	2.36
Pelvis Tilt	2.015	2.150	0.135	1.962	2.128	0.166	−6.70	−8.47
Trunk Rotation	3.507	2.001	−1.506	3.368	1.907	−1.461	42.93	43.38
Hip Flexion Extension	1.857	1.859	0.002	1.561	1.643	0.083	−0.12	−5.29
Trunk Tilt	3.507	2.001	−1.506	3.368	1.907	−1.461	42.93	43.38
Pelvis Rotation	2.015	2.150	0.135	1.962	2.128	0.166	−6.70	−8.47
Shoulder Flexion Extension	16.615	30.109	13.494	13.689	29.364	15.675	−81.21	−114.51
Ankle Dorsiflexion Plantarflexion	1.349	3.979	2.630	1.030	3.116	2.086	−194.93	−202.60
Pelvis Obliquity	2.015	2.150	0.135	1.962	2.128	0.166	−6.70	−8.47

RMSE: root mean square error; MAD: mean absolute difference.

**Table 3:** Subject-Level Pre- to Post-Training Changes in Kinematic Gait Parameters.

Parameter	*p*-Value	Cohen’s d *	95% CI
Trunk Lateral Lean (deg)	0.0002	−1.35	[−5.94, −2.39]
Ankle Dorsi/Plantarflexion (deg)	0.0081	0.83	[0.82, 4.47]
Knee Flexion/Extension (deg)	0.0252	0.68	[0.32, 4.07]
Trunk Rotation (deg)	0.6193	−0.14	[−2.60, 1.61]
CoM AP Excursion (m)	0.1840	−0.38	[−2.16, 0.46]
Shoulder Flexion/Extension (deg)	0.1182	0.45	[−5.99, 47.14]
Pelvis Obliquity (deg)	0.8369	−0.06	[−4.28, 3.53]
Pelvis Tilt (deg)	0.5401	−0.17	[−1.13, 0.62]
Pelvis Rotation (deg)	0.6281	0.13	[−1.67, 2.67]
CoM ML Sway (m)	0.2043	−0.36	[−2.44, 0.57]
CoM Vertical Oscillation (m)	0.9341	−0.02	[−0.06, 0.05]
Hip Flexion/Extension (deg)	0.6508	0.12	[−3.67, 5.67]
Trunk Tilt (deg)	0.8281	0.06	[−1.13, 1.39]

* Effect sizes are reported as paired Cohen’s d with 95% confidence intervals. Statistical significance was set at *p* < 0.05. Healthy control data were used exclusively as a normative reference and were not included in inferential statistical analyses.

**Table 4:** Spatiotemporal and CoM Gait Parameters Before and After Movement Training.

Parameter	Pre-Training μ ± Sd	Post-Training μ ± Sd	Control Group μ
Stride Time (s)	1.13 ± 0.119	1.27 ± 0.18	1.11
Cadence (Steps/Min)	107.05 ± 9.76	95.90 ± 12.79	111.20
Speed (m/s)	1.07 ± 0.36	0.91 ± 0.30	1.27
Stride Length (m)	1.19 ± 0.37	1.15 ± 0.35	1.41
Step Width (m)	0.19 ± 0.18	0.21 ± 0.17	0.16
Stance (Right) (%)	55.97 ± 11.68	59.71 ± 6.60	60.81
Swing (Right) (%)	44.03 ± 11.68	40.29 ± 6.60	39.19
Double Support (%)	24.26 ± 0.3	25.47 ± 6.94	24.48
Arm Swing Rom (Deg)	11.33 ± 0.12	5.49 ± 0.03	21.93
Trunk Lean Rom (Deg)	5.79 ± 1.4	3.36 ± 1.26	0.01
CoM Vertical Osc (mm)	0.03 ± 0.04	0.03 ± 0.000	0.03
CoM Mediolateral Sway (m)	0.15 ± 0.03	0.23 ± 0.02	0.14
CoM Anterior-Posterior Excursion (m)	0.47 ± 0.15	0.47 ± 0.01	1.42

## Data Availability

The data supporting the findings of this study are available from the corresponding author upon reasonable request. Publicly available data from the COMPWALK-ACL dataset were also used in this study (https://doi.org/10.5281/zenodo.15624356, accessed on 7 June 2025).

## Abbreviations

The following abbreviations are used in this manuscript:

JIA	Juvenile Idiopathic Arthritis
CoM	Center of Mass
ROM	Range of Motion
RMSE	Root Mean Square Error
MAD	Mean Absolute Difference
IMU	Inertial Measurement Unit
SD	Standard Deviation
ACL	Anterior Cruciate Ligament
AP	Anterior–Posterior
ML	Mediolateral
